# Interaction between the barley allelochemical compounds gramine and hordenine and artificial lipid bilayers mimicking the plant plasma membrane

**DOI:** 10.1038/s41598-018-28040-6

**Published:** 2018-06-28

**Authors:** Simon Lebecque, Jean-Marc Crowet, Laurence Lins, Benjamin M. Delory, Patrick du Jardin, Marie-Laure Fauconnier, Magali Deleu

**Affiliations:** 1 TERRA—AgricultureIsLife, Gembloux Agro-Bio Tech, University of Liège, Gembloux, Belgium; 2 Laboratory of Molecular Biophysics at Interfaces, Gembloux Agro-Bio Tech, University of Liège, Gembloux, Belgium; 3 Ecosystem Functioning and Services, Institute of Ecology, Leuphana University, Universitätsallee 1, 21335 Lüneburg, Germany; 4 Laboratory of Plant Biology, Gembloux Agro-Bio Tech, University of Liège, Gembloux, Belgium; 5 General and Organic Chemistry Laboratory, Gembloux Agro-Bio Tech, University of Liège, Gembloux, Belgium

## Abstract

Some plants affect the development of neighbouring plants by releasing secondary metabolites into their environment. This phenomenon is known as allelopathy and is a potential tool for weed management within the framework of sustainable agriculture. While many studies have investigated the mode of action of various allelochemicals (molecules emitted by allelopathic plants), little attention has been paid to their initial contact with the plant plasma membrane (PPM). In this paper, this key step is explored for two alkaloids, gramine and hordenine, that are allelochemicals from barley. Using *in vitro* bioassays, we first showed that gramine has a greater toxicity than hordenine towards a weed commonly found in northern countries (*Matricaria recutita* L.). Then, isothermal titration calorimetry was used to show that these alkaloids spontaneously interact with lipid bilayers that mimic the PPM. The greater impact of gramine on the thermotropic behaviour of lipids compared to hordenine was established by means of infrared spectroscopy. Finally, the molecular mechanisms of these interactions were explored with molecular dynamics simulations. The good correlation between phytotoxicity and the ability to disturb lipid bilayers is discussed. In this study, biophysical tools were used for the first time to investigate the interactions of allelochemicals with artificial PPM.

## Introduction

For decades, the use of synthetic herbicides in agriculture has been raising concerns about public health and environmental preservation^1–3^. Additionally, herbicides could become increasingly less effective as weeds develop resistance to the commonly used products^4^. For these reasons, new ways to support efficient and sustainable crop production are being explored. Allelopathy, defined as any direct or indirect harmful or stimulatory effect by one plant (including microorganisms) on another through production of chemical compounds (called allelochemicals) that escape into the environment^5^, is one of these alternative ways that might lead to a reduction in the amount of synthetic herbicides used in the field.

Farmers may take advantage of allelopathy in several ways^6,7^. The ability to produce and release allelochemicals could become a selection trait in order to obtain allelopathic cultivars able to reduce the spreading of weeds^8,9^. The impact of practices such as allelopathic crop residue management on weed development is another topic of interest in current agricultural research^10,11^. Another potential application of allelopathy is based on the use of allelochemicals as new herbicides or as leads for new herbicides^12,13^.

**Figure 1:**
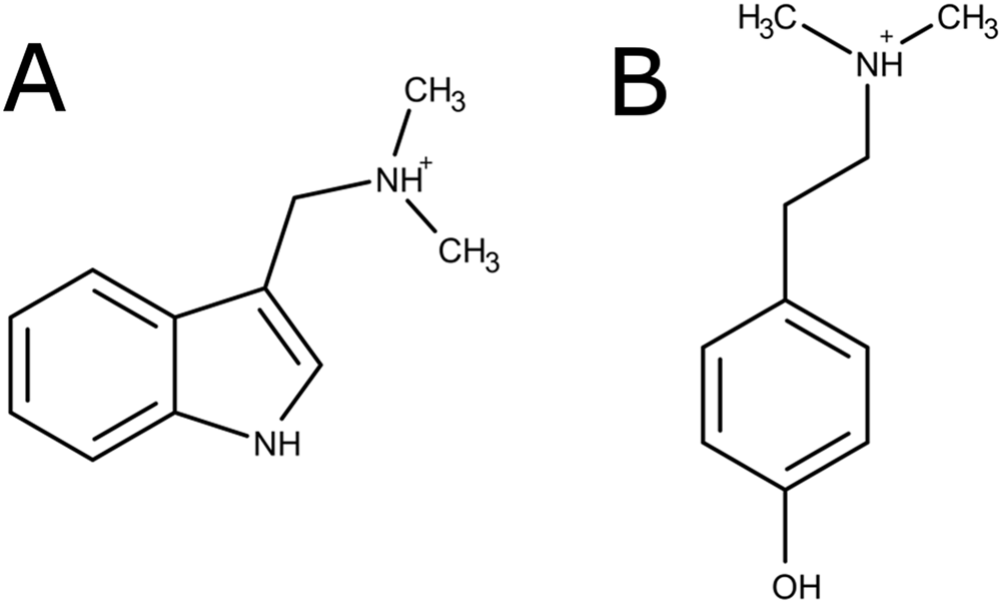
Molecular structures of the alkaloids under investigation. (**A**) Gramine and (**B**) hordenine (both in the protonated state).

Regardless of the way in which allelopathy is to be exploited, the identification of allelochemicals and a thorough understanding of their modes of action are needed for safe and efficient use of these new approaches. Hence, recent decades have seen an increasing interest in those fields that led to the identification of numerous allelopathic species, from which various potential allelochemicals were extracted and identified. Barley, being both a major cereal grain and a well-known allelopathic plant^14^, was no exception to the rule, and more than 40 compounds are now listed as candidate allelochemicals from this species^15^. A large number of these chemicals are small aromatic compounds; for example, gramine and hordenine (see Fig. 1 for structures) are alkaloids that have been extensively studied by Lovett *et al*.^16–18^. The authors shed light on their phytotoxicity against white mustard and gave new insights into production levels as a function of plant cultivar and plant age.

Recently, more importance has been given to the study of gramine compared to hordenine. In addition to its phytotoxicity, gramine has been reported to be toxic to mammals^19,20^, insects^21,22^, bacteria^23^ and fungi^24,25^. More recently, publications have emphasized its potential as an algicide^26,27^. This broad-spectrum toxicity suggests that the effects of gramine might be mediated by more than one mode of action and/or through action on ubiquitous targets. Liu and Lovett described the effect of gramine on the root tip ultrastructure of white mustard^16^. Organellar disorganization, the occurrence of lipid droplets and increases in both size and number of vacuoles were the general symptoms that the authors observed. Several publications focused on the effect of gramine on either isolated subcellular components^28,29^ or complete unicellular organisms^30^. They showed that effects on energy metabolism could play a role in gramine toxicity. Subsequent studies of gramine-induced toxicity in algae highlighted an enhancement of oxidative stress by the allelochemical that might be responsible for its algicidal effect^26,27^.

However, little attention was given to the initial contact between these small aromatic compounds and the plasma membrane, which is a prerequisite to any toxic mechanism. In this paper, we explore the interactions of gramine and hordenine with artificial plant plasma membranes. We first describe the phytotoxicity induced by these alkaloids on *Matricaria recutita* L. (chamomile), a common weed in northern countries. Then, we highlight the molecular interactions between the allelochemicals and model membranes by using isothermal titration calorimetry (ITC) and infrared spectroscopy. Finally, we propose a structure – function relationship with regard to the ability to interact with membranes based on molecular dynamics (MD).

## Results

### Phytotoxicity assays

The phytotoxicity of gramine and hordenine was evaluated by measuring the root length of *M. recutita* seedlings grown in the presence or absence of the alkaloids. Table 1 shows the mean root length of *M. recutita* for each treatment after 7 days. It can be seen that hordenine exhibits a slight but significant phytotoxic effect on *M. recutita*, as revealed by a decrease in mean root length of approximately 20% compared to the control. Treatments, including gramine, are much more effective in reducing the root growth in chamomile, with an inhibition percentage reaching more than 70% for the 1 mM gramine treatment. The concentration-induced differences are not significant according to the statistical analysis. One can also note that the mean root length for both gramine and hordenine together at 0.5 mM is not significantly different from that for 0.5 mM gramine alone. This should exclude the existence of any synergistic effect between hordenine and gramine on *M. recutita* at these concentrations.

**Table 1:** Phytotoxicity of gramine and hordenine. Effects of various gramine and hordenine treatments on the mean root length of *M.* *recutita* seedlings (treatments that do not share a common letter are significantly different, Tukey’s test with α = 0.05)

Treatment	Mean root length (±SD) (mm)	Inhibition percentage
Control	22.6^a^ (±2.6)	
Hordenine 0.5 mM	17.8^b^ (±1.1)	21.2%
Hordenine 1 mM	16.4^b^ (±2.3)	27.5%
Gramine 0.5 mM	8.4^c^ (±1.7)	62.9%
Gramine 1 mM	6.2^c^ (±1.1)	72.6%
Gramine 0.5 mM + Hordenine 0.5 mM	8^c^ (±1.2)	64.5%

.

### Ability of alkaloids to insert into lipid bilayers

**Figure 2:**
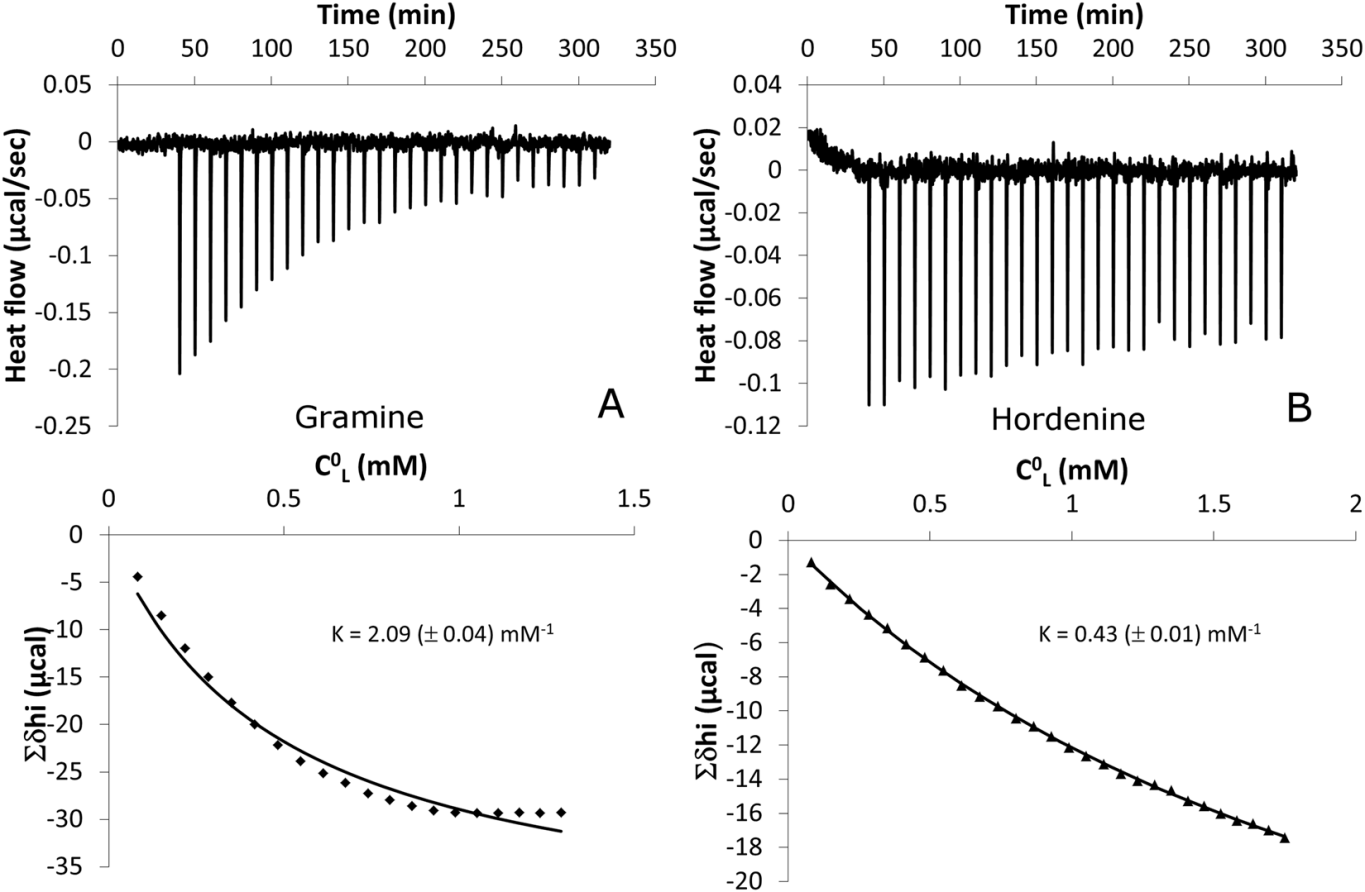
Interactions between alkaloids and liposomes evidenced by ITC experiments. Upper panels: raw data from ITC experiments. Each peak corresponds to a single injection of 10 µL of a 10 mM PLPC-PLPG (molar ratio 4:1) LUV suspension into a solution containing (**A**) 10 µM gramine and (**B**) 10 µM hordenine at 26 °C. LUV suspension and alkaloid solutions were buffered at pH 6.15 with MES – NaOH. Lower panels: cumulative heat of binding (Σδhi) as a function of the lipid concentration in the cell (C0L). The solid line corresponds to the best fit using eq. 1. From this fit, the partition coefficient K is determined for each alkaloid (fitting uncertainty in brackets).

For insight into the possible interactions between the alkaloids and a model plant plasma membrane, biophysical studies were carried out. Isothermal titration calorimetry experiments have been performed to study the ability of gramine and hordenine to insert into large unilamellar vesicles (LUVs) composed of 1-palmitoyl-2-linoleoyl-sn-glycero-3-phosphocholine (PLPC) and 1-palmitoyl-2-linoleoyl-sn-glycero-3-phospho-rac-(1-glycerol) (PLPG) (4:1 molar ratio). Figure 2 (upper panels) shows typical raw data from an ITC experiment for gramine (A) and hordenine (B). The negative and decreasing heat flow observed after each injection of liposomes into a 10 µM alkaloid solution indicate that both gramine and hordenine spontaneously interact with lipids. Figure 2 (lower panels) also displays the corresponding cumulative heat of binding (Σδhi) plotted against the lipid concentration in the cell (C^0^_L_). By curve fitting to this data set, it is possible to determine the partition coefficient K for each alkaloid^31,32^. According to these K values, the affinity of gramine for the lipid bilayer is greater than that of hordenine.

### Effect of gramine and hordenine on lipid phase transition temperature

**Figure 3:**
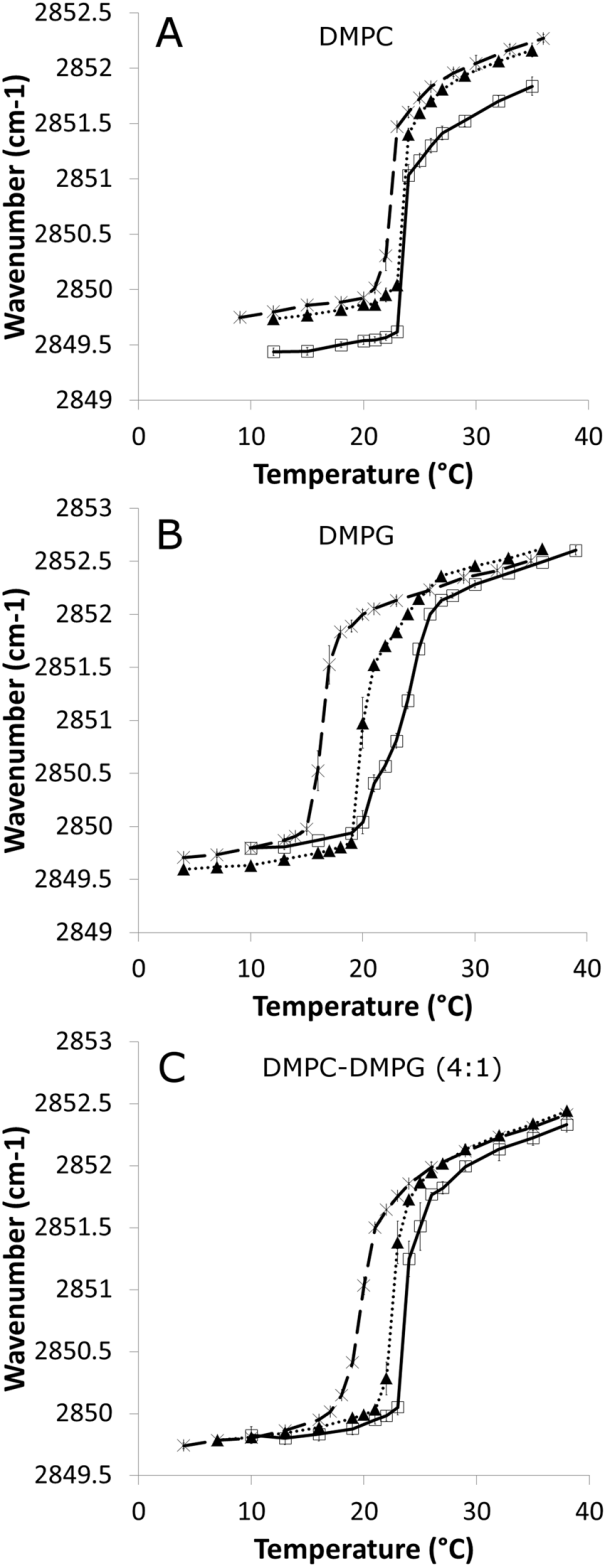
Effects of alkaloids on phase transition temperature of liposomes. Evolution of _s_ν_CH2_ as a function of temperature for (**A**) DMPC liposomes, (**B**) DMPG liposomes and (**C**) DMPC-DMPG (molar ratio 4:1) liposomes in the absence of alkaloids (solid line, ) or in the presence of gramine (dashed line, ×) or hordenine (round dotted line, ▲).

To study the impact of gramine and hordenine on lipid bilayer properties, Fourier transform infrared spectroscopy (FTIR) experiments were performed. When applied to a liposomal suspension, infrared spectroscopy gives an interesting insight into the acyl chain conformation of lipids composing the liposomes. Peaks at approximately 2850 cm^−1^ and 2920 cm^−1^ on IR spectra for lipid samples correspond to the methylene symmetric (_s_ν_CH2_) and antisymmetric (_as_ν_CH2_) stretching vibrations, respectively^33^. These IR bands are sensitive to acyl chain conformation, and their maxima will shift to higher values as the relative amount of gauche conformers increases. This feature allows monitoring of the lipid phase transition that occurs in a lipidic system at a given temperature. Indeed, during chain melting, lipidic systems undergo a transition from an ordered gel phase where lipid acyl chains are in an all-trans conformation to a disordered liquid crystalline phase in which the number of gauche conformers is increased. On a graph plotting the wavenumber of the peak maximum corresponding to the methylene symmetric stretching vibration against temperature, the phase transition is thus translated into an increase in wavenumber. Panels A, B and C from Fig. 3 illustrate this for a system composed of pure 1,2-dimyristoyl-sn-glycero-3-phosphocholine (DMPC), pure 1,2-dimyristoyl-sn-glycero-3-phospho-rac-(1-glycerol) (DMPG) and mixed DMPC:DMPG (molar ratio 4:1) in the presence or absence of alkaloids. Table 2 summarizes the phase transition temperatures (Tm) calculated for each system from the curves shown in Fig. 3A–C as described above. Figure 3A shows that the phase transition temperature of DMPC vesicles is slightly reduced when gramine is added. The gramine-induced decrease is approximately 1 °C (see Table 2). No T_m_ change is observed when hordenine is added to DMPC multilamellar vesicles (MLVs).

**Table 2:** Phase transition temperature of the different systems as determined from FTIR measurements (fitting uncertainty in brackets). ∆Tm was computed for each system in the presence of an alkaloid with respect to the same system in the absence of the alkaloid

Lipid composition	Alkaloid	Phase transition temperature (°C)	∆Tm
DMPC	/	23.69 (±0.15)	
DMPC	Gramine	22.6 (±0.18)	−1.09
DMPC	Hordenine	23.65 (±0.13)	−0.04
DMPG	/	23.67 (±0.15)	
DMPG	Gramine	16.54 (±0.17)	−7.13
DMPG	Hordenine	20.52 (±0.25)	−3.15
DMPC:DMPG (4:1)	/	24.03 (±−0.21)	
DMPC:DMPG (4:1)	Gramine	20.17 (±0.21)	−3.86
DMPC:DMPG (4:1)	Hordenine	22.81 (±0.17)	−1.22

.

From Fig. 3B, it can be seen that gramine and hordenine have a strong effect on the phase transition temperature of DMPG vesicles, with Tm reductions of approximately 7.1 and 3.1 °C, respectively. When working with mixed liposomes (DMPC and DMPG in a molar ratio 4:1), both alkaloids affect the lipid phase transition temperature, as illustrated in Fig. 3C. In such systems, gramine is again responsible for a larger decrease in Tm than hordenine (3.9 vs. 1.2 °C).

### Interactions with lipid bilayer: a molecular dynamics insight

**Figure 4:**
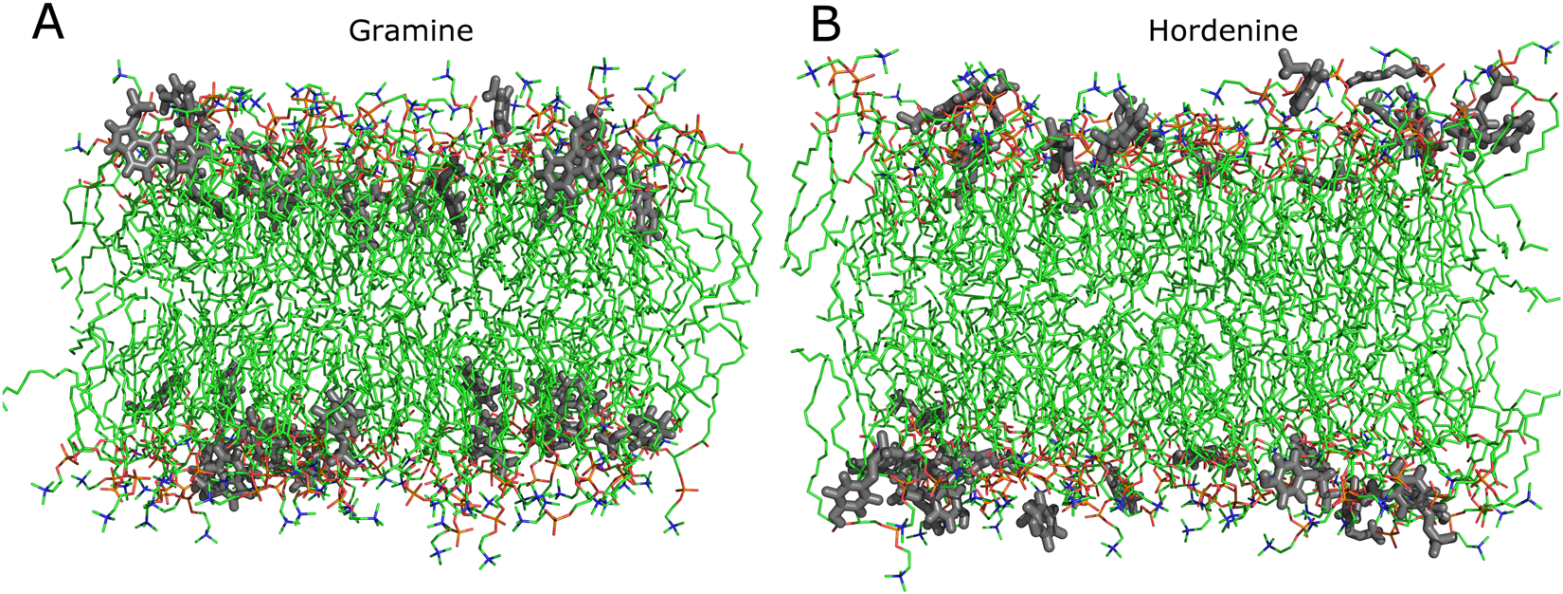
Snapshots after 500 ns simulations of a PLPC bilayer with alkaloids. (**A**) 128 PLPC bilayer with 32 gramine molecules and (**B**) 128 PLPC bilayer with 32 hordenine molecules. For the sake of clarity, water molecules and ions are omitted. Grey: alkaloid molecules, green: carbon atoms, red: oxygen atoms, orange: phosphor atoms, blue: nitrogen atoms.

Molecular dynamics allows for the analysis of the behaviour of the alkaloids in the presence of lipid bilayers at the molecular level. MD simulations were thus performed to investigate the molecular mechanisms that underlie alkaloid insertion into lipid bilayers and their subsequent interactions. Initially, 32 gramine or hordenine molecules were placed outside a lipid bilayer (composed of 128 PLPC or PLPG molecules, thus reaching a lipid:alkaloid ratio of 4:1 as in the FTIR experiments). After 500 ns of simulation, most of the alkaloids were inserted into the bilayer as illustrated in Fig. 4, in agreement with ITC assays.

**Figure 5:**
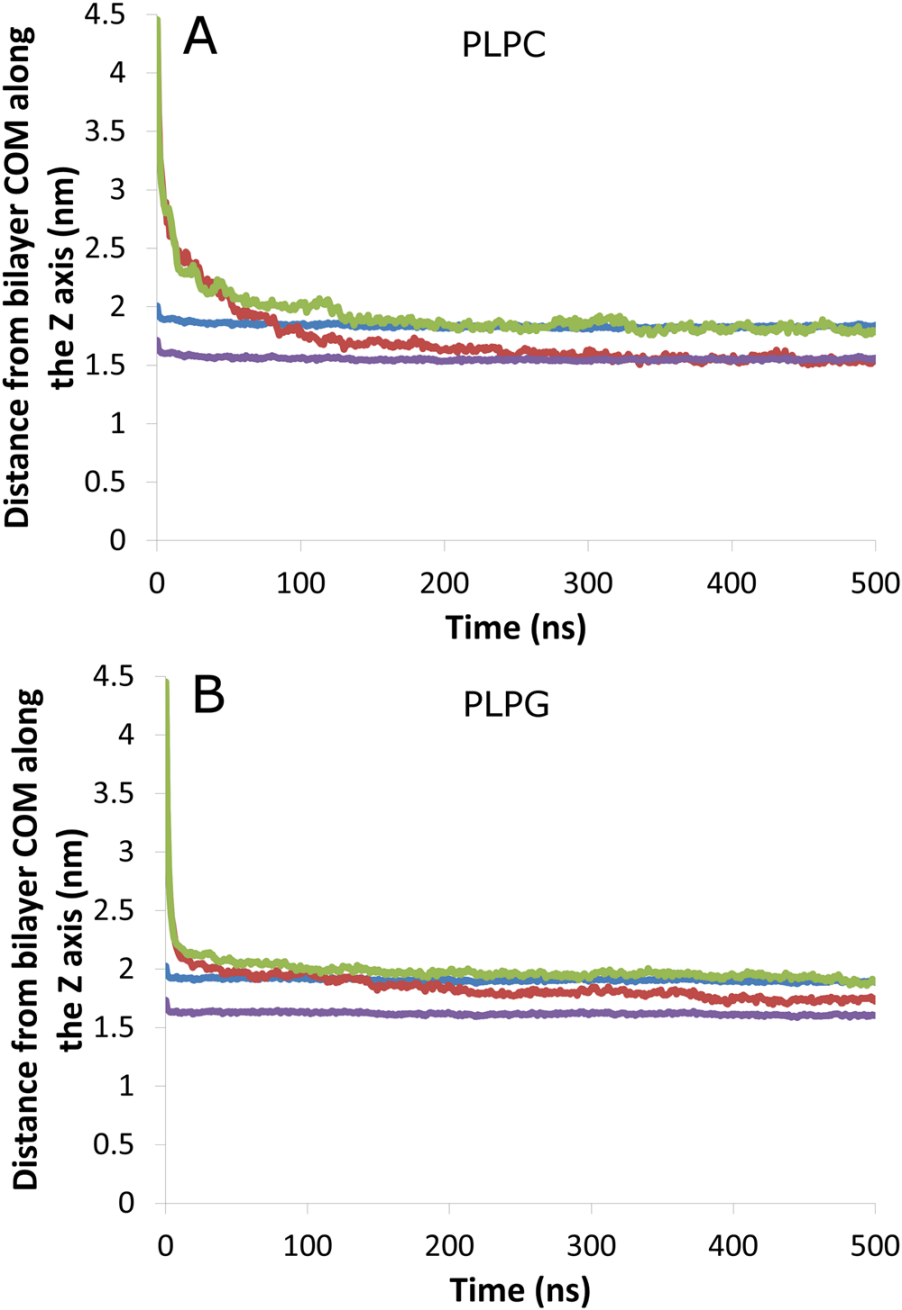
Location of gramine and hordenine within the lipid bilayers. Evolution of the distance from the (**A**) PLPC bilayer centre of mass and (**B**) PLPG bilayer centre of mass along the Z-axis (nm). Gramine: red line, hordenine: green line, phosphorus atoms: blue line, glycerol backbone COM: violet line.

Figure 5 A shows the average distance between the alkaloid centre of mass (COM) and the COM of the PLPC bilayer composed along the Z-axis. For better visualization, the distance between P atoms from PLPC headgroups and the bilayer COM along the Z axis is also displayed, as is the distance between the glycerol backbone COM and the bilayer COM. It can be observed that both alkaloids adsorb to the bilayer surface in approximately 100 ns. Thereafter, gramine molecules penetrate deeper into the bilayer approximately at the glycerol level, while hordenine molecules seem to be preferentially located at the water-lipid interface. Figure 5B gives the same information for a system containing a 128 PLPG bilayer. The first contact between the alkaloids and the PLPG bilayer seems to occur significantly faster than with the zwitterionic bilayer. This is most likely due to electrostatic attraction from the negatively charged phosphate groups that are not compensated in PLPG, contrary to PLPC molecules that also bear a positively charged choline group. It can also be seen that even though gramine is again more deeply buried than hordenine, as in the PLPC bilayer, it does not penetrate the PLPG bilayer as deeply as it does the PLPC bilayer.

**Figure 6:**
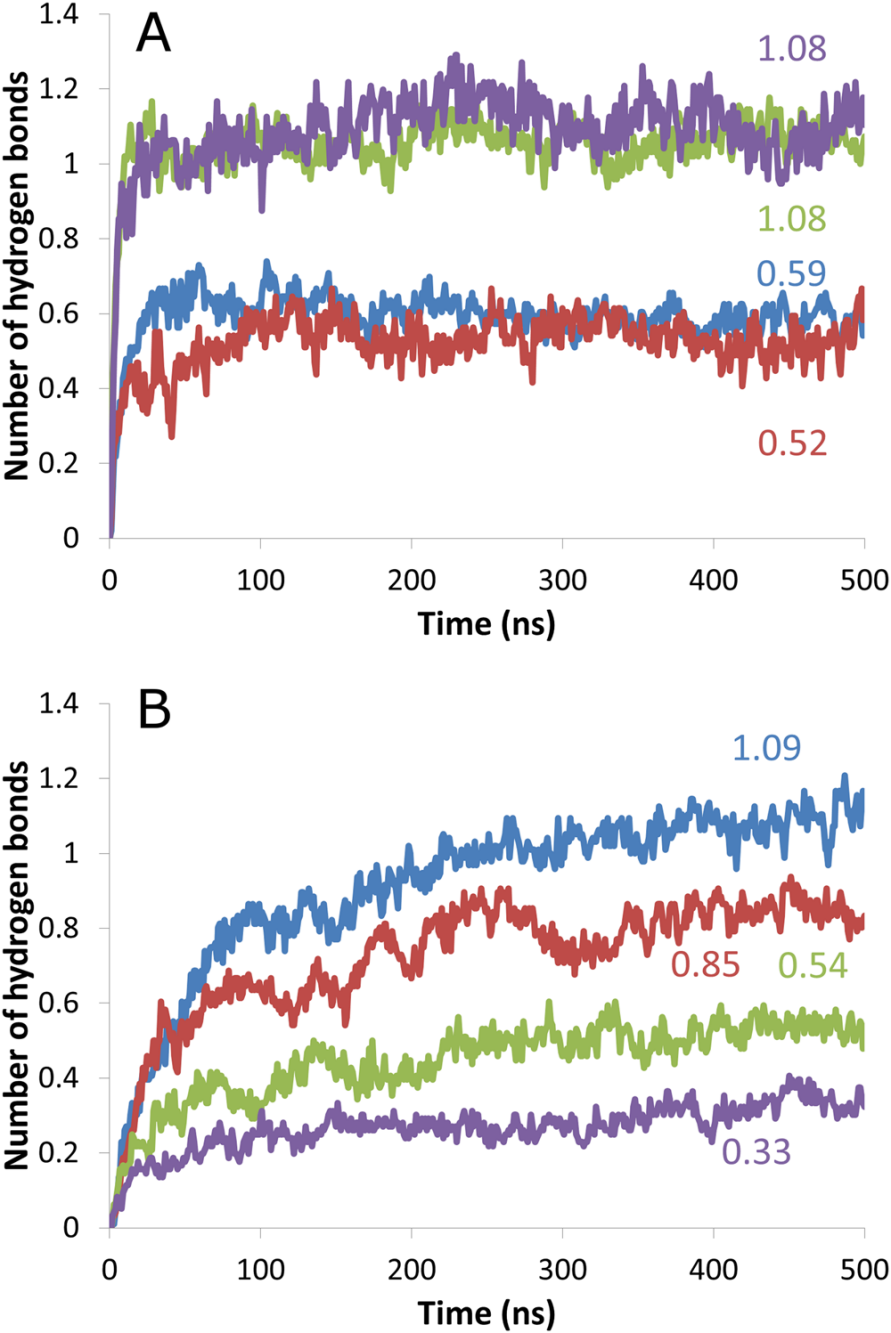
Analysis of hydrogen bond formation between lipids and alkaloids. Time evolution of the number of hydrogen bonds (**A**) between alkaloids and lipid phosphate groups and (**B**) between alkaloids and lipid ester groups (average number per alkaloid in both cases). PLPC – gramine: blue line; PLPC – hordenine: red line; PLPG – gramine: green line; PLPG – hordenine: violet line. For each alkaloid – lipid pair, the average number of hydrogen bonds per alkaloid during the last 100 ns is displayed.

Again, this more superficial location is probably due to electrostatic interactions with negatively charged phosphate groups that are more accessible in PLPG. As a result, the number of hydrogen bonds between gramine molecules and phosphate groups is much higher in PLPG systems than in PLPC systems, as shown in Fig. 6A. The same is observed for hordenine (Fig. 6A). The average number of hydrogen bonds per alkaloid during the last 100 ns of simulation is also displayed for each alkaloid-lipid pair. As shown in Fig. S1 (see supplementary data), the overall hydrogen bond number between alkaloids and lipids is only marginally affected by the lipid species. In both systems, gramine establishes more hydrogen bonds than hordenine, although the difference is moderate. In addition to phosphate groups, alkaloids can also create H bonds with the ester groups of the lipid molecules. The evolution of such H bonds is plotted against time in Fig. 6B. Given the previous considerations, it is not surprising to find a much higher number of alkaloid – ester H bonds when the bilayer is made of PLPC molecules. It is noteworthy that in a given system, gramine establishes more H bonds with ester groups than hordenine. The small discrepancies between the number of total H bonds (Fig. S1) and the sum of the contributions (Fig. 6A,B) from phosphate and ester groups observed for PLPG systems come from a negligible number of H bonds between the alkaloids and the headgroup glycerol.

**Figure 7:**
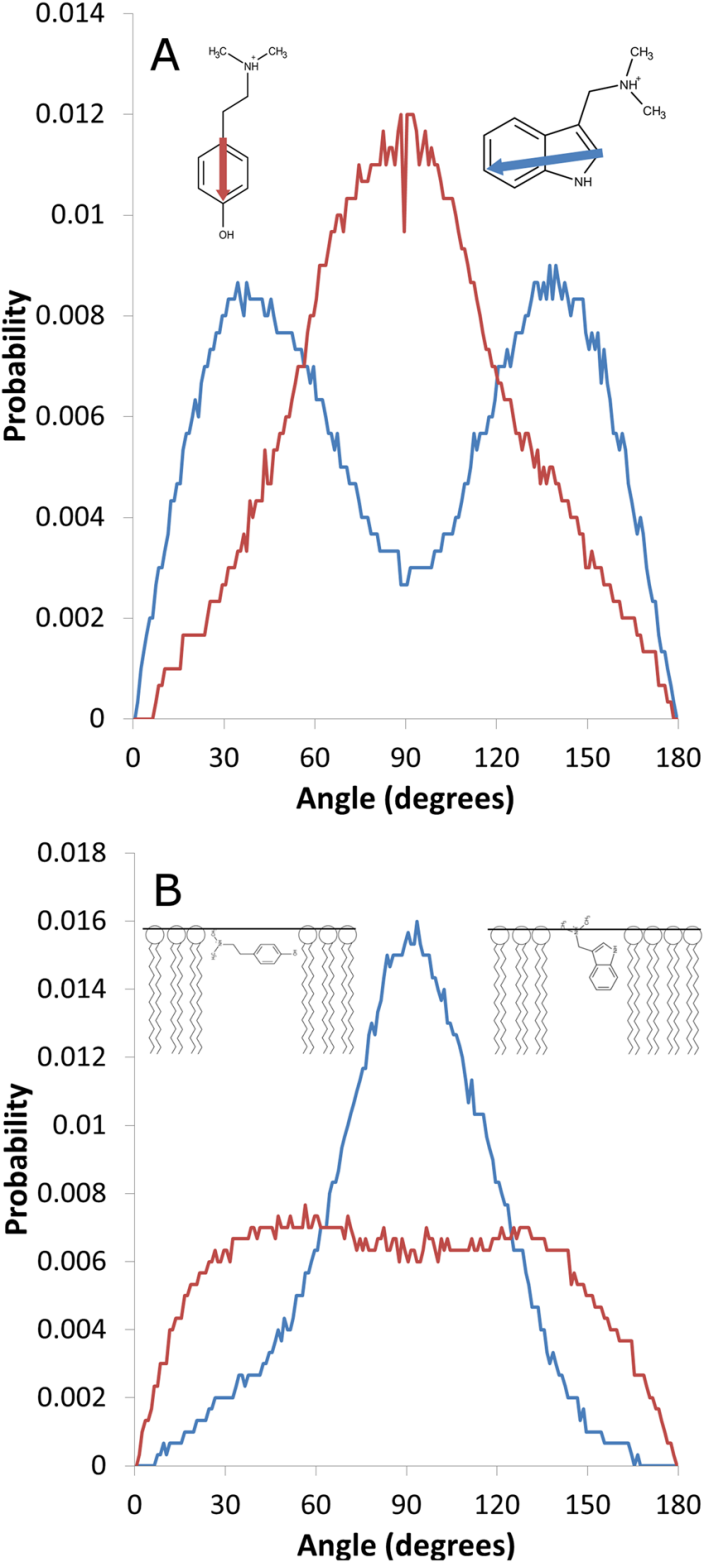
Orientation of gramine and hordenine within the PLPC bilayer. Distribution of angles (**A**) between the PLPC bilayer normal and vectors defined on each alkaloid as shown in the insets and (**B**) between the PLPC bilayer normal and vectors orthogonal to the surface defined on each alkaloid by 3 points of the cyclic part (insets of **B**: schematic representation of the hordenine (left) and gramine (right) orientation within the lipid bilayer). Blue line: gramine, red line: hordenine.

Polar interactions can involve two groups in each alkaloid. For gramine, these groups are the protonated amine function and the nitrogen from the indole cycle, while for hordenine, the protonated amine and the phenol function should be involved. The position of these groups within the molecular structure of each alkaloid could lead to differences between gramine and hordenine with respect to their orientation within the lipid bilayer. Figure 7A displays the distribution of angles calculated between a vector orthogonal to the PLPC bilayer (referred to as the bilayer normal) and vectors defined on each alkaloid, as shown in the insets. For hordenine, the distribution is unimodal and centred at approximately 90°, which means that the vector is roughly perpendicular to the bilayer normal and thus parallel to the bilayer plane. In contrast, the distribution of angles for gramine is bimodal. Bimodality is explained by the presence of alkaloids within both leaflets of the bilayer, which leads to two populations of supplementary angles from a unique orientation. The gramine vector forms an angle of approximately 35° with the bilayer normal, which means that the insertion of gramine into the bilayer is much more “vertical”. Figure 7B shows the distribution of angles between the PLPC bilayer normal and a vector orthogonal to the surface defined on each alkaloid from 3 points of the cyclic part. The unimodal distribution centred at approximately 90° observed for gramine confirms that the indolic cycle is buried almost perpendicularly to the bilayer surface. The much broader angle distribution observed for hordenine suggests that the orientation of the aromatic cycle oscillates between two extreme angles of approximately 30 and 150°. The data shown in Fig. 7 for PLPC are very similar to the data for PLPG (see Fig. S2, supplementary data).

## Discussion

In this paper, we studied the toxicity of gramine and hordenine towards *M. recutita*, a common weed, and explored the interactions of these molecules with model plant plasma membranes. Both alkaloids were shown to be phytotoxic, with gramine being much more efficient in decreasing the mean root length than hordenine. The toxicity observed for gramine is in agreement with previous studies^14,16^. From Liu and Lovett’s work^16^, it could be inferred that hordenine is as phytotoxic as gramine, as the compounds reduce the mean root length of white mustard by a similar order of magnitude. However, Overland^14^ had previously described the inability of hordenine to inhibit the growth of *Stellaria media* and its slight ability to inhibit flowering. Our results are somewhere in between these opposite trends, with hordenine being able to significantly affect the mean root length of chamomile, but to a lesser extent than gramine.

In addition, while Liu and Lovett concluded that gramine and hordenine have synergistic effects in inhibiting white mustard root length, our data do not support the existence of any such synergy. The contradiction between the studies may have various origins, such as the difference in target species. In addition, Liu and Lovett did not buffer their gramine and hordenine solutions, while in our assays, the solutions were buffered at pH 6.15. Differences in pH may generate differences in the protonation state of the alkaloids, which could have an impact on their toxicity. Finally, the high phytotoxicity of the 0.5 mM gramine treatment alone could eclipse synergistic effects that would occur with less concentrated solutions.

To investigate the initial contact that these alkaloids would have with the target plant cells and how they could interact with the plasma membrane, we used various biophysical techniques. By means of isothermal titration calorimetry experiments, we have shown that gramine and hordenine spontaneously interact with model plant plasma membranes, with gramine having a higher affinity for lipids. Infrared spectroscopy allowed us to study the impact of gramine and hordenine on the thermotropic behaviour of lipids, while MD enabled us to shed light on potential molecular mechanisms that underlie these effects.

For ITC experiments and MD simulations, PLPC and PLPG were used to study the specific interactions of alkaloids with these lipids that are representative of plant plasma membranes (PPMs). Indeed, phospholipids are a major component of PPMs, and among phospholipids, PC is the main lipid species^34^ (and references cited therein). Negatively charged PG represents up to 14% of phospholipids and has been further selected since total negatively charged lipids can reach a ratio of 30% of phospholipids^34^ (and references cited therein). The acyl chain length and saturation level were chosen because palmitic and linoleic acids are the most common fatty acids in phospholipids from PPMs^34^ (and references cited therein). For IR experiments, DMPC and DMPG were used because PLPC and PLPG have phase transition temperatures well below 0 °C, making their study highly inconvenient. This is not expected to have any impact on the qualitative effects described in the paper, as the polar headgroups remain unchanged.

The phase transition temperatures found for pure DMPC and pure DMPG are in very good agreement with the literature^35–37^. The fact that the phase transition temperature of mixed DMPC-DMPG MLVs is slightly higher than the Tm of either of the pure lipid components has also been reported previously^38^.

Since the impact of both alkaloids on the lipid phase transition temperature is much higher in the DMPG system than in the DMPC system, it is assumed that their effect is mainly mediated through interactions with the lipid polar moiety rather than with the lipid hydrophobic core. Indeed, DMPC and DMPG have the same acyl chains and only differ at the headgroup (phosphatidylcholine and phosphatidylglycerol, respectively). This is in good agreement with MD simulations, in which alkaloids were localized at a depth between phosphate groups and glycerol groups.

For given acyl chains, polar headgroups determine how tightly packed the lipids are in their gel phase. Their size, their charge and their ability to form H bonds between each other are parameters that affect the stabilization of the gel phase^35^. This is directly linked to the lipid phase transition temperature, as a more stabilized gel phase will need higher temperatures to undergo melting^35^. Hence, what favours tight packing of a lipid species will increase its Tm, as is the case for divalent cations added to PG vesicles, for instance^36^. In this case, the repulsive electrostatic interactions between the phosphate groups are screened by the divalent cations that also behave as bridges between headgroups. In the present study, we have shown that the DMPG Tm is decreased when gramine or hordenine is present, which is symptomatic of membrane disturbance. Even though the alkaloids are positively charged and might hence partially screen the negative charges carried by the phosphate groups, they seem to exert a destabilizing effect, possibly arising from H bond interference. McMullen *et al*.^39^ suggested that attractive forces that occur between polar headgroups in the DMPC bilayer and in the DMPG bilayer should be very similar since both lipids have approximately the same Tm. However, the authors assumed that the nature of the headgroup - headgroup interactions should differ. Repulsive electrostatic interactions in DMPG are thought to be overcompensated by intermolecular H bonds. DMPC molecules, however, do not possess a H bond donor, and only weak electrostatic interactions occur between phosphate groups and choline moieties. All in all, the strength of the interactions between polar headgroups is supposed to be similar, resulting in a Tm of approximately 23 °C for both lipid species. According to our MD data, gramine and hordenine are able to establish H bonds with phosphate groups much more efficiently in a PG bilayer than in a PC bilayer, which is attributed to the absence of a positively charged group in PG. Our hypothesis is that these alkaloid – lipid headgroup H bonds weaken the lipid headgroup – headgroup H bonds and, together with the electrostatic attraction occurring between alkaloids and lipid phosphate groups, promote effective alkaloid intercalation between the headgroups. This could increase the headgroup – headgroup spacing and thus loosen the packing, destabilizing the bilayer and resulting in a Tm decrease.

For PC, the presence of the positively charged choline groups prevents the protonated alkaloids from interacting with phosphate groups as much as they do in PG systems. H bond data from MD suggest that both gramine and hordenine are more likely to interact with ester groups rather than phosphate groups in PC systems, while the opposite is observed with PG. Additionally, the native absence of any headgroup – headgroup H bonds in PC means that none of the alkaloids can act on them to lower Tm. However, gramine was shown to be oriented almost perpendicularly to the bilayer surface, while hordenine is roughly parallel to the bilayer surface. Given the orientation and location of gramine, it is reasonable to think that its polar moiety is favourably located at the lipid-water interface, while the most apolar atoms are more deeply buried to avoid contact with water. We assume that this vertical anchoring into the bilayer is responsible for minor steric disturbances around the glycerol region that give rise to the slight reduction in Tm for DMPC. On the other hand, for hordenine, the polar moiety – apolar moiety frontier is less evident, with an OH group and an amine group located on opposite sides of the aromatic ring. Hence, vertical anchoring is not thermodynamically favoured, as it would involve the location of a polar group in a less polar environment. As a result, hordenine is less able to perturb the DMPC bilayer than gramine, and no Tm shift is observed in the DMPC – hordenine system. This is also translated into a higher number of hydrogen bonds between gramine and ester groups for a given lipid species. This difference in orientation might also explain the higher efficiency of gramine in lowering the Tm of DMPG vesicles compared to hordenine.

Interactions with membranes could play a role in gramine and hordenine phytotoxicity. Even though additional evidence is needed, the good correlation between the ability to lower Tm and the observed phytotoxicity might be meaningful in this regard. The molecular effects of the alkaloids on lipid bilayers explored in this study are instructive in several ways. Subtle changes in lipid properties may have a direct impact on the conformation of proteins that are embedded within the lipid bilayer through lateral pressure modifications. This kind of mechanism has been frequently cited to explain the mode of action of small amphipathic compounds, such as anaesthetics, for instance^40–42^. This hypothesis surely deserves attention since many putative allelochemicals are small aromatic compounds bearing polar group(s), such as benzoic and cinnamic acid derivatives^43,44^. In the case of gramine and hordenine, these considerations might be specifically extended to the thylakoids and mitochondria. Thylakoid membranes contain 5 to 15% PG^45,46^ plus 10 to 30% SQDG (sulfoquinovosyl diacylglycerol), another negatively charged lipid. In the dimeric PSII complex, the PG ratio increases to 30% and is proven to play a major role in electron transport mediated by plastoquinone Q_B_^46^. Mitochondrial membranes are made of approximately 5% PG and more than 10% cardiolipin^47^, a lipid species with a molecular structure very similar to a dimer of PG. These considerations may be related to previous studies that reported an impact of gramine on energy metabolism^28–30^.

Apart from the putative mechanism of action through a direct effect on lipid properties, our data suggest an attractive effect of the lipids on gramine and hordenine together with a preferential location around the polar headgroups. This result could be a clue suggesting that if the alkaloids have specific targets, these targets might be located in membranes as well. In addition, it leads to the question “how do those alkaloids enter the cell?”, as they mostly interact with polar parts of lipids. Over the course of the three MD replicates of each system (1.5 µs per system in total), none of the 32 alkaloids crossed the PLPC or the PLPG bilayer. Even though the sampling is not sufficient to rule out spontaneous membrane crossing, this observation might indicate that simple diffusion is not sufficient to ensure the transport of the protonated alkaloids through the lipid bilayer. Their uptake might thus depend on active transport (with “pumps” that require metabolic energy) or facilitated diffusion (with carrier or channel proteins that do not require metabolic energy). Since the structure of gramine is similar to that of tryptophan and the structure of hordenine is similar to that of tyrosine, amino acid transporters^48^ might be involved in such uptake mechanisms. Moreover, it has been shown that antifungal azole drugs accumulate in fungi cells by facilitated diffusion^49,50^, and a similar mechanism might occur here.

Finally, the molecular behaviour described in this paper might have consequences on human health. Recently, dopamine has been reported to lower DMPG Tm without having a significant effect on DMPC Tm^51^. Serotonin has been described as being able to decrease the phase transition temperature of DMPC^52–54^. In addition to their structural similarity, dopamine and serotonin share other similarities with hordenine and gramine, respectively, in their effects on the thermotropic behaviour of lipids. This comparison strengthens our own biophysical results, as similar molecules have similar effects on lipids. However, it also means that the alkaloids investigated here might have noxious effects on mammals through neurotransmitter interference, as already suspected for gramine and other indolealkylamines^19^. This does not imply a straightforward decision as to whether these alkaloids should be used in weed management or not. However, it calls for more research on the toxicity, bioaccumulation and biodegradability of these compounds. Studies on the transport of gramine and hordenine from the roots to other parts of plant would also be of interest, as it would give valuable information about their *in planta* mode of action and their availability for animals. It is worth remembering that such precautions should concern not only gramine and hordenine but also every product potentially used in agricultural fields.

Biophysical techniques such as the ones used in this paper could be useful for highlighting membrane-mediated modes of action of phytotoxic compounds, including already used herbicides. It would be valuable to determine if such interactions with lipid bilayers can also be correlated to deleterious effects on human health. These still unexplored topics surely deserve attention, as they can lead to a better understanding of toxicity mechanisms.

## Methods

### Plant material and chemicals

*M. recutita* seeds were purchased from Emorsgate Seeds, Norfolk, United Kingdom.

DMPC, PLPC and PLPG were purchased from Avanti Polar Lipids, Inc. All other chemicals, including DMPG, gramine and hordenine, were purchased from Sigma Aldrich.

### Phytotoxicity assays

One repetition for one treatment was performed as follows. Seventy-five *M. recutita* seeds were placed on Whatman filter paper wetted with 3 mL of solution. Solutions were prepared from 5 mM MES buffer in distilled water adjusted to pH 6.15 by adding NaOH. This buffer alone was used as the control, while gramine and/or hordenine were added at different concentrations to obtain different treatments (namely, hordenine 0.5 mM, hordenine 1 mM, gramine 0.5 mM, gramine 1 mM and gramine 0.5 mM + hordenine 0.5 mM). Each filter paper together with the seeds was placed in a Petri dish sealed with Parafilm®. All Petri dishes were then placed in a growth chamber with a photoperiod of 16:8 h, a light intensity of 250 lux and a temperature of 25 °C for 7 days. Thereafter, the Petri dishes were scanned with a resolution of 1200 dpi. The total root length of each seedling was then measured from the scans with the software ImageJ and used as a phytotoxicity marker.

All experiments were carried out using a randomized block experimental design, with a block being composed of 7 Petri dishes (5 treatments + 2 controls performed in a random order). In total, 7 blocks were analysed, resulting in 7 repetitions for each treatment and 14 repetitions for the control. Statistical analyses were undertaken using Minitab 17.3 software.

### Lipid preparation and isothermal titration calorimetry

For isothermal titration calorimetry experiments, large unilamellar vesicles were prepared as follows. Small amounts of lipids (PLPC:PLPG, 4:1 molar ratio) were dissolved into chloroform-methanol (2:1) in a round-bottom flask. The solvent was removed under low pressure by a rotary evaporator, and the flask was then kept overnight under vacuum to remove solvent traces. The lipid film was then hydrated with 5 mM MES buffer prepared from Milli-Q water with a pH adjusted to 6.15 by adding NaOH. The flask was maintained at a temperature (~37 °C) well above the transition phase temperature of the lipids for at least 5 minutes and then vortexed for 1–2 minutes. This cycle was repeated 5 times. Thereafter, the MLV suspension underwent 5 freeze-thaw cycles. To obtain LUVs, the MLV suspension was then extruded 11 times through polycarbonate filters with a pore diameter of 100 nm.

ITC measurements were performed with a VP-ITC from Microcal. The sample cell (volume: 1.4565 mL) contained alkaloid solution from the same buffer as the LUV suspension, and its temperature was maintained at 26 °C. Small volumes of the LUV suspension were successively injected into the sample cell: a first injection of 2 µL (not taken into account for data treatment) was followed by 28 injections of 10 µL. A spacing time of 600 s was used between each injection. Origin 7.0 software was used for data treatment, following a previously described method^31,32^ in which the cumulative heats of binding are fitted to the equation:

1 $$\sum _{k=1}^{i}\delta {h}_{k}={\rm{\Delta }}{H}_{D}^{w\to b}{V}_{cell}{C}_{A}^{0}\frac{K{C}_{L}^{0}}{1+K{C}_{L}^{0}}$$

where δh_k_ is the heat produced after each injection, ∆H_D_^w→b^ is the difference in molar enthalpy originating from the transfer of the alkaloid molecules from the aqueous phase to the bilayer membrane, V_cell_ is the volume of the sample cell, C_A_^0^ and C_L_^0^ are the total alkaloid and lipid concentrations, respectively, in the cell after i injections and K is the partition constant.

### Lipid preparation and Fourier transform infrared spectroscopy

For FTIR experiments, MLVs were prepared as follows. Five milligrams of lipids (pure DMPC, pure DMPG or DMPC:DMPG mix at a 4:1 molar ratio) was dissolved into chloroform-methanol (2:1) in a round-bottom tube. When present, alkaloids were dissolved together with the lipids to reach a lipid:alkaloid molar ratio of 4:1. Solvent was evaporated with a gentle N_2_ flux, and the tube was then kept overnight under vacuum to remove solvent traces. The lipid film was then hydrated with 150 µL of 50 mM MES buffer prepared from Milli-Q water with the pH adjusted to 6.15 by adding NaOH. The tube was maintained at a temperature well above the transition phase temperature of the lipids for at least 5 minutes and then vortexed for 1–2 minutes. This cycle was repeated 5 times. Thereafter, the MLV suspension underwent 5 freeze-thaw cycles.

FTIR measurements were performed with a Bruker Equinox 55 spectrometer. Sixty µL of the prepared suspension were squeezed between CaF_2_ windows separated by a 50 µm Teflon spacer. The windows were part of an infrared cell assembled and placed in a thermojacket linked to a thermostated bath. The cell was cooled down and kept at the starting temperature for 15 minutes before a first measurement was made. The temperature was then increased (at an approximate rate of 1 °C/min) and the sample was allowed to equilibrate for 10 minutes at each desired temperature before spectrum recording. Temperature intervals between two measurements were 3, 2 or 1 °C, depending on how close the phase transition temperature was. Every spectrum was the result of an average of 64 scans between 4000 cm^−1^ and 400 cm^−1^ with a resolution of 2 cm^−1^.

The spectra were corrected for CO_2_ and H_2_O absorption and baseline corrected. Peak picking was made by a standard method from OPUS software. The values of peak maximum corresponding to the symmetric stretching of methylene groups for each temperature were then averaged for 3 replicates and plotted against temperature. A Boltzmann curve was fitted to the resulting sigmoidal curve with Origin 7.0 software to obtain its inflection point, which gave the phase transition temperature of the system.

### Molecular dynamics

Simulations have been performed with GROMACS 4.6.7 and the united atom GROMOS 53a6 force field^55^ with three replicates. Gramine and hordenine topologies have been manually refined from Automatic Topology Builder’s results^56^. PLPC and PLPG topologies were derived from POPC and POPG topologies developed by Piggot *et al*. as Gromos-CKP forcefield^57,58^. Bilayers containing 128 lipids were generated and hydrated by using Memgen^59^ and then allowed to equilibrate during 200 ns simulations before use. Each system was solvated with SPC water^60^. 32 alkaloids were randomly added at least 1 nm away from the bilayer surface and 32 Cl^−^ ions were added to get an overall charge of zero. All systems firstly underwent a 100 ps NVT equilibration followed by a 1 ns NPT equilibration during which alkaloids were under position restraints. 500 ns production runs were then performed. For the production runs, temperature was maintained to an average value of 298 K by using the Nose-Hoover thermostat^61,62^ with a τ_T_ = 0.5 ps. Semi-isotropic pressure (1 bar) was maintained by using the Parrinello-Rahman barostat^63^ with a compressibility of 4.5 × 10^−5^ bar^−1^ and τ_P_ = 2 ps. Electrostatic interactions were treated by using the particle mesh Ewald (PME) method. A twin-range cut-off was used for Van der Waals interactions (short-range = 0.9 nm, long-range = 1.4 nm). Bond lengths were maintained with the LINCS algorithm^64^. Trajectories were analyzed with GROMACS tools as well as with homemade scripts and were visually analyzed with VMD^65^ and PYMOL^66^ software packages.

## Electronic supplementary material

Supplementary information
