# Supplementary material for: Interaction between the barley allelochemical compounds gramine and hordenine and artificial lipid bilayers mimicking the plant plasma membrane
Source: Sci Rep. 2018 Jun 28;8:9784. doi: 10.1038/s41598-018-28040-6 (PMC6023908; doi:10.1038/s41598-018-28040-6)
Supplement: Supplementary file 1 — Supplementary information [file 41598_2018_28040_MOESM1_ESM.pdf]

Supplementary Information

Interaction between the barley allelochemical compounds gramine and hordenine and artificial lipid bilayers mimicking the plant plasma membrane

Simon Lebecque, Jean-Marc Crowet, Laurence Lins, Benjamin M. Delory, Patrick du Jardin, Marie-Laure Fauconnier, Magali Deleu

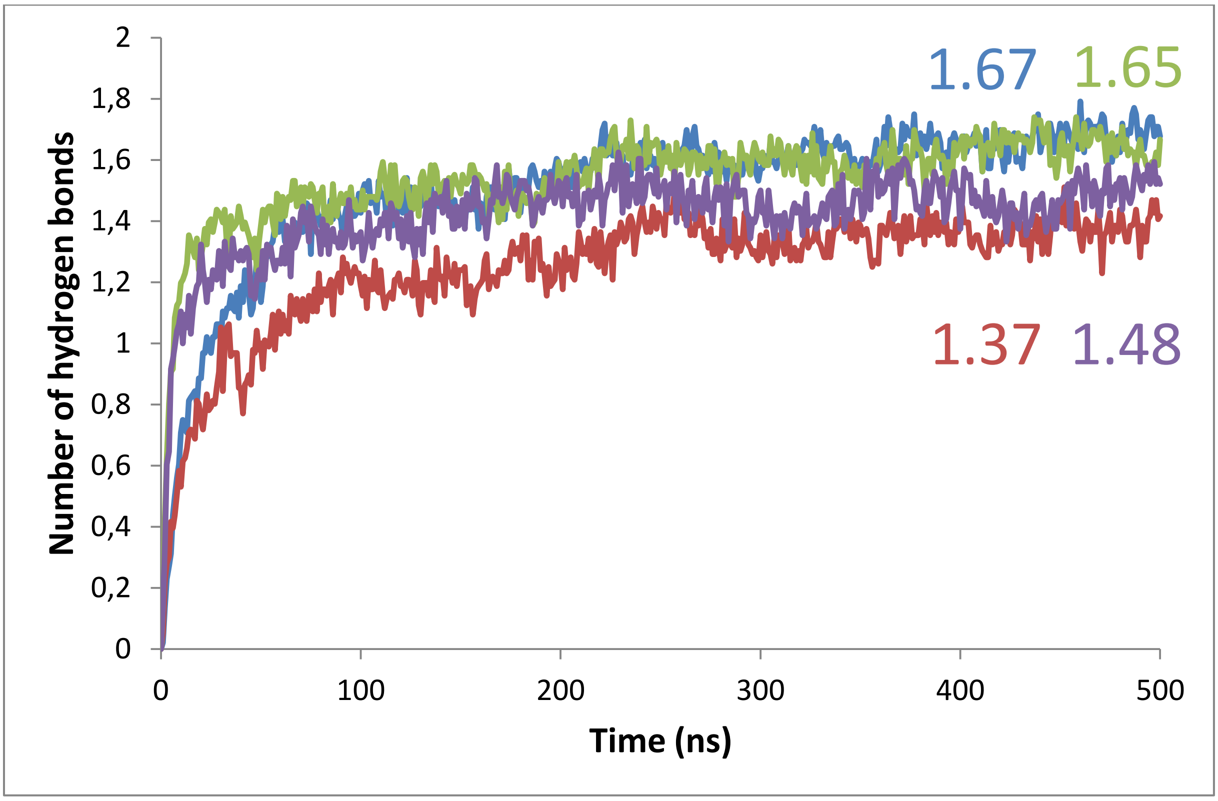

Figure S1. Time evolution of the number of hydrogen bonds between alkaloids and lipids (average number per alkaloid). PLPC – gramine: blue line; PLPC – hordenine: red line; PLPG – gramine: green line; PLPG – hordenine: violet line. For each alkaloid – lipid pair, the average number of hydrogen bonds per alkaloid during the last 100 ns is displayed.

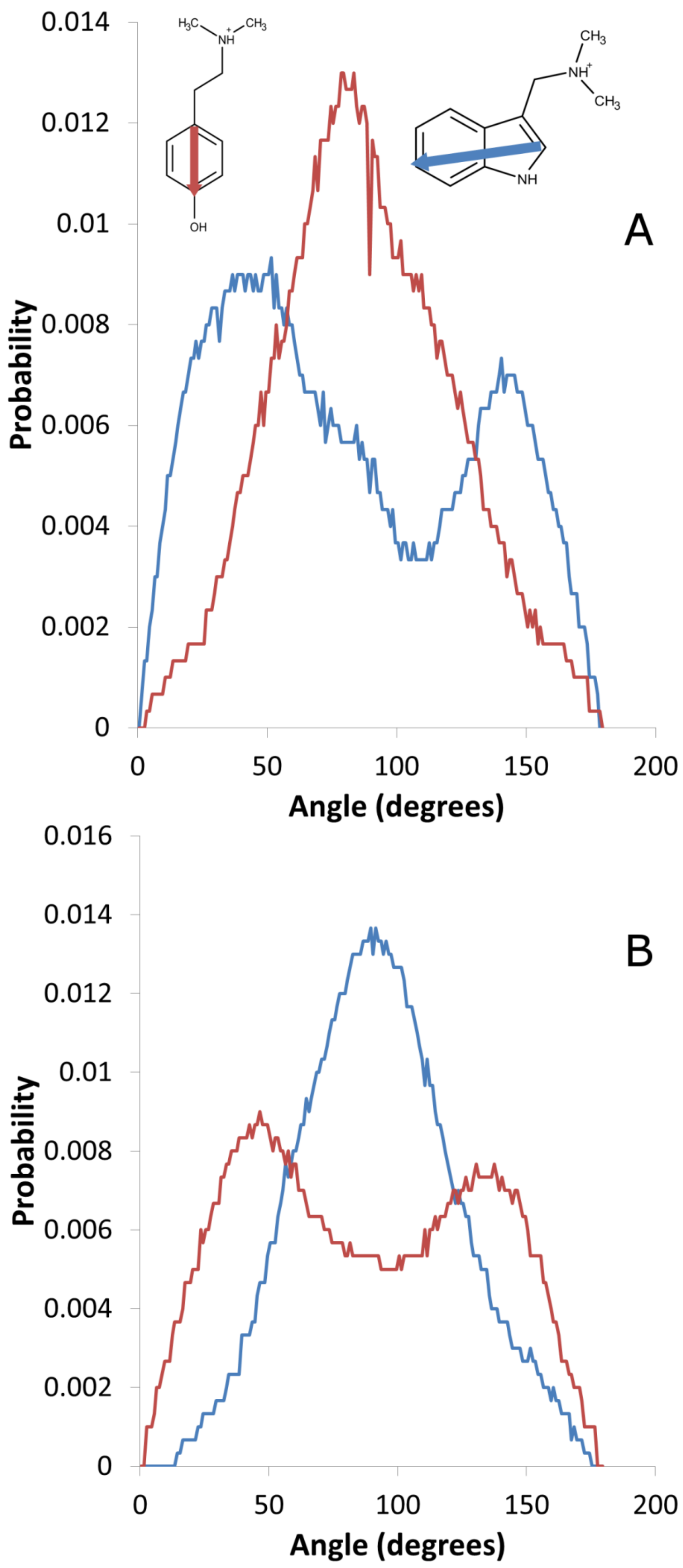

Figure S2. Distribution of angles (A) between the PLPG bilayer normal and vectors defined on each alkaloid as shown in the insets and (B) between the PLPG bilayer normal and vectors orthogonal to the surface defined on each alkaloid by 3 points of the cyclic part. Blue line: gramine, red line: hordenine.
